# Prevalence of G6PD deficiency and submicroscopic malaria parasites carriage in malaria hotspot area in Northwest, Tanzania

**DOI:** 10.1186/s12936-023-04801-1

**Published:** 2023-12-07

**Authors:** Alphaxard Manjurano, Eric Lyimo, Coleman Kishamawe, Justin Omolo, Jacklin Mosha, Miyaye Donald, Paul Kazyoba, Saidi Kapiga, John Changalucha

**Affiliations:** 1 Mwanza Centre, National Institute for Medical Research, Mwanza, Tanzania; 2 Mabibo Centre, National Institute for Medical Research, Dar es Salaam, Tanzania; 3 Mwanza Intervention Trials Unit, National Institute for Medical Research, Mwanza, Tanzania; 4 Department of Infectious Disease Epidemiology, London School of Hygiene and Tropical Medicine, London, UK

**Keywords:** G6PD deficiency, MDA, Primaquine, Malaria, Submicroscopic *Plasmodium falciparum*

## Abstract

**Background:**

The use of primaquine for mass drug administration (MDA) is being considered as a key strategy for malaria elimination. In addition to being the only drug active against the dormant and relapsing forms of *Plasmodium vivax*, primaquine is the sole potent drug against mature/infectious *Plasmodium falciparum* gametocytes. It may prevent onward transmission and help contain the spread of artemisinin resistance. However, higher dose of primaquine is associated with the risk of acute haemolytic anaemia in individuals with a deficiency in glucose-6-phosphate dehydrogenase. In many *P. falciparum* endemic areas there is paucity of information about the distribution of individuals at risk of primaquine-induced haemolysis at higher dose 45 mg of primaquine.

**Methods:**

A retrospective cross-sectional study was carried out using archived samples to establish the prevalence of G6PD deficiency in a malaria hotspot area in Misungwi district, located in Mwanza region, Tanzania. Blood samples collected from individuals recruited between August and November 2010 were genotyped for G6PD deficiency and submicroscopic parasites carriage using polymerase chain reaction.

**Results:**

A total of 263 individuals aged between 0 and 87 were recruited. The overall prevalence of the X-linked G6PD A− mutation was 83.7% (220/263) wild type, 8% (21/263) heterozygous and 8.4% (22/263) homozygous or hemizygous. Although, assessment of the enzymatic activity to assign the phenotypes according to severity and clinical manifestation as per WHO was not carried out, the overall genotype and allele frequency for the G6PD deficiency was 16.4% and 13. 2%, respectively. There was no statistically significant difference in among the different G6PD genotypes (p > 0.05). Out of 248 samples analysed for submicroscopic parasites carriage, 58.1% (144/248) were *P. falciparum* positive by PCR. G6PD heterozygous deficiency were associated with carriage of submicroscopic *P. falciparum* (*p* = 0.029).

**Conclusions:**

This study showed that 16.4% of the population in this part of North-western Tanzania carry the G6PD A− mutation, within the range of 15–32% seen in other parts of Africa. G6PD gene mutation is widespread and heterogeneous across the study area where primaquine would be valuable for malaria control and elimination. The maps and population estimates presented here reflect potential risk of higher dose of primaquine being associated with the risk of acute haemolytic anaemia (AHA) in individuals with a deficiency in glucose-6-phosphate dehydrogenase and call further research on mapping of G6PD deficiency in Tanzania. Therefore, screening and education programmes for G6PD deficiency is warranted in a programme of malaria elimination using a higher primaquine dose.

## Background

The current renewed effort for malaria elimination is aimed to spot hotspot areas to identify individuals having gametocytes for mass drug administration (MDA) [1–3]. Studies have indicated that sub-microscopic malaria carriage is common in the population [4–6]. Also, it is well documented that individuals carrying sub-microscopic parasites are the cause of seasonal malaria transmission [7]. The use of primaquine for mass drug administration is being considered as an option for parasite clearance to block transmission of *Plasmodium falciparum* in areas approaching malaria elimination [8, 9].

Universal efforts to attain malaria elimination have recently lead the World Health Organization (WHO) to recommend for a single dose of 0.25 mg/kg primaquine as a *P. falciparum* gametocytocide [10]. Furthermore, in areas of low malaria transmission, the WHO has recommended the inclusion of primaquine 0.75 mg base/kg (adult dose 45 mg) to artemisinin-based combination therapy (ACT) regimens for falciparum malaria [11]. However, there is a concern of using primaquine due to the haemolysis associated with the drug in Glucose-6-Phosphate Dehydrogenase (G6PD) deficiency individuals [12]. Severity of haemolysis depends on the *G6PD* variant present, gender, ingestion of some foods (for example, fava beans) as well as dose and duration of primaquine exposure, causing haemolysis which sufficiently requires blood transfusion [13].

G6PD deficiency is an X-linked genetic disorder and about 400 million people worldwide have a deficiency of this enzyme [14]. G6PD is highly polymorphic, at least 186 mutations have been characterized in the G6PD gene [15], although not all are polymorphic and of clinical significance. The variants of G6PD include: the common G6PD B (wild type), G6PD A (non-deficient type) and G6PD A− (deficient type). Both G6PD A and G6PDA− differ from G6PD B by a variation at nucleotide 376 (A→G), and at the same time G6PD A− had an extra mutation at nucleotide 202 (G→A) [16, 17], the latter being predominant in sub-Saharan Africa. The wild-type B variant and A+ variant (which carries a single mutation at nucleotide 376), have normal or near-normal enzyme activities. With an additional mutation at nucleotide 202, the A− variant has approximately 12% of the wild type enzyme activity [18] and is normally associated with mild haemolysis. Other common G6PD deficiencies include: Mediterranean and Asia types, the differences related to the mutation’s position on chromosome and geographic locations [19, 20].

The enzyme G6PD protects against the oxidative stress of haemolytic anaemia induced by both infection and anti-malarials [21]. Despite the haemolytic stress associated with G6PD deficiency there are also reports of protection to malaria afforded by the enzyme deficiency [19, 22].

Studies have shown that asymptomatic *P. falciparum* infection is common in the population [4, 23, 24], and G6PD deficient individuals may be carrying malaria parasites without symptoms [25]. It is globally recognized that novel tools and strategies are required to eliminate foci of residual *P. falciparum* malaria transmission [26]. Primaquine is the only approved anti-malarial drugs used to eliminate hypnozoites of *Plasmodium vivax* and *P. falciparum* gametocytes [27–29]. Some studies on tolerance of primaquine undertaken in G6PD deficient individuals have suggested that, courses of lower dosage treatment as described by Hill [30–32] may be safe. However, safety concern remains related to the acute haemolytic anaemia associated with a higher primaquine doses which will be required to clear the radical *P. vivax* and *Plasmodium ovale* in G6PD deficient individuals [33, 34]. Therefore, given the wide genetic and phenotypic variability of G6PD, it is considered as priority to carry out phenotypic and genotypic analysis in different populations where primaquine MDA is being considered for elimination [35–37].

Misungwi district located in Mwanza region in Northwest Tanzania is an area with moderately malaria transmission; the overall prevalence of *P. falciparum* infection in the area is estimated to be 31.4% [38] and that the district is a malaria hotspot area [5, 23, 39]. A study carried out in the area, found that on average, 50% of household members are parasite carriers within hotspots [23]. Due to more frequent infections, these people have acquired higher levels of immunity to clinical malaria than those living outside the hotspots. As a consequence, they can remain asymptomatic for long periods of time, while acting as infectious reservoirs for onward transmission [4, 23]. It has been shown that submicroscopic carriage is common in different malaria transmission settings [4] and that submicroscopic *P. falciparum* gametocyte carriage is not uncommon in an area of low and seasonal malaria transmission and is a source of maintaining malaria transmission [40, 41], a campaign which requires primaquine dosing strategy to reduce haemolysis in individuals with G6PD deficiency. However, policy makers have been reluctant to implement this recommendation due to primaquine safety concerns and a lack of data on its efficacy [42].

While malaria transmission declines across to a great extent of its range and the likelihood of elimination is gradually more considered [43]; Misungwi district in Mwanza, North west Tanzania has been previously reported to be a malaria hotspot area and the results suggested that community-wide MDA, instead of screen and treat strategies, may be needed to successfully treat the asymptomatic, subpatent parasite reservoir and reduce transmission [23]. However, there is no studies which has been carried out assessing the frequency of G6PD deficiency. Therefore, there is a need to establish the distribution of G6PD deficiency in malaria hotspot area and identify individuals who may be carrying the malaria parasites (submicroscopic parasites) for mass drug administration for clearing of the parasites in individuals which may be a source of transmission. This study was carried out to map the distribution of G6PD deficiency in malaria hotspot area in Mwanza region located in North-western Tanzania which is important in resource poor-settings for future malaria elimination.

## Methods

### Study area

The study was conducted in Misungwi district (latitude 2.85000 S, longitude 33.08333 E) is located in Mwanza region, the northwest of Tanzania at an altitude of 1178 m above sea level, the district is situated 60 km from Mwanza town. The district has a moderate level of malaria transmission (meso-endemic). The district has two annual rainy seasons, the long rains between February and May and the short rains between November and December. The dry and relatively hot season is June to September. Transmission intensity has a seasonal cycle, with peaks in malaria incidence 1 to 2 months after the rains start. The prevalence of malaria infection in the region is estimated to be 31.4% by microscopy during a Demographic and Health Survey (DHS).

### Study design

A retrospective cross-sectional study using archived samples for establishing the prevalence of G6PD deficiency in a malaria hotspot area was carried out in Misungwi district located in Mwanza region, Northwest of Tanzania.

### Study population and sampling techniques

**Fig. 1:**
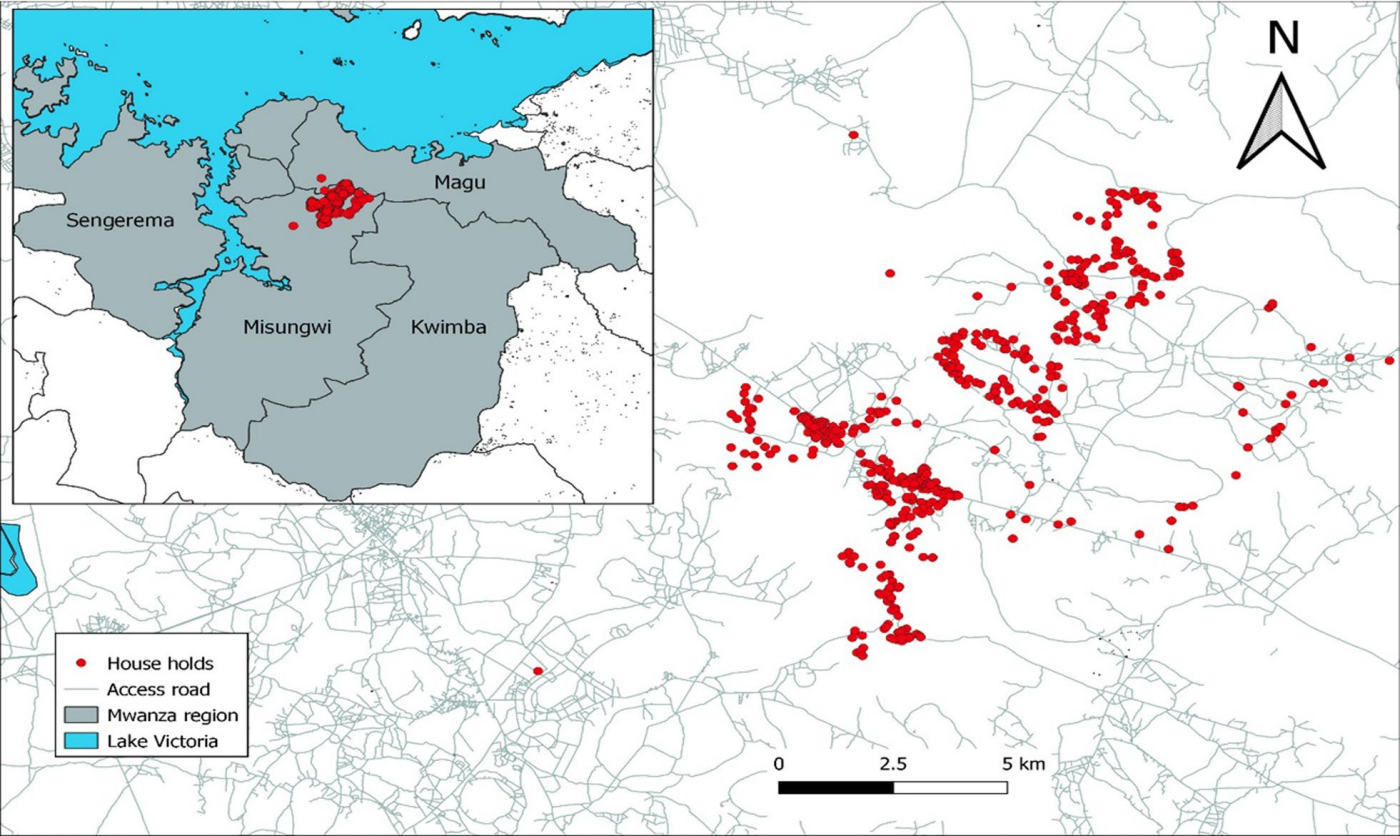
Location of the study site within Tanzania and distribution of households included in the study (red points) showing local topography and road network (grey lines) (Adapted from Mosha et al. [23])

The study utilized blood samples collected from individuals living in four villages (Fig. 1) from August to November 2010 [23] who questionnaires were used to collect demographic and clinical information of the study participants. Any subject who reported a fever within the previous 24 h was tested for malaria using a histidine-rich protein 2 (HRP2) malaria rapid diagnostic test (RDT, *Paracheck*-*Pf*®, Orchid Biomedical Systems, Goa, India) and referred to a study clinician for management of their febrile illness as reported by Mosha et al. [23]. In this study, a purposive and random sampling was employed. Briefly, selection of archived filter paper blood samples from individuals in four villages in a single ward located in Northern part of Misungwi district was carried out and a random selection of samples from individuals in each village was used. However, individual presented with fever and RDT positive were excluded. Several studies have reported the prevalence of G6PD deficiency varies between 5 and 32.5% in malaria-endemic areas of Africa and Asia, and its geographical distribution overlaps with the distribution of malaria. The sample size calculation was based on G6PD deficiency prevalence of 11% in a lowland area carried out in Tanzania [44]. Hence, a minimum of 250 individuals was enough to be included in this study. For this study 263 study participants were recruited and provided blood specimen for G6PD deficiency assessment whereas a total of 248 samples from the same individuals were used for assessment of submicroscopic parasite carriage. Finger prick blood samples were collected on Standard 3MM Whatman filter paper, dried overnight at room temperature. The samples were individually sealed into plastic bags and stored with desiccant at − 20 °C.

### Sample processing and DNA extraction

Sample processing and the laboratory analyses were performed at the National Institute for Medical Research (NIMR), Mwanza Research Centre laboratory where DNA was extracted from filter papers using the QIAamp DNA Mini Kit (QIAGEN, UK), following manufacturer’s instructions.

### G6PD deficiency genotyping (the G6PD A− allele (202G→A)

G6PD deficiency was genotyped by allele-specific PCR and followed by gel electrophoresis [27]. Briefly, G6PD deficiency variants (B, A and A−) was determined by a simple high-throughput method using PCR amplification with forward and reverse primers, 5′CTGGCCAAGAAGAAGATCTACC 3′ and 5′GAAAAACGCAGCAGAGCACAG 3′ respectively. DNA was amplified in a total reaction volume of 30 µl consisting of reaction buffer, 2 mM MgCl_2_, 0.1 µmol of each primer, 0.1 mM dNTPs each and 1 U of BioTaq DNA polymerase (Bioline). A touchdown programme was used to prevent non-specific amplification; an initial denaturation step at 95 °C for 5 min was followed by 14 cycles at 95 °C for 40 s, 68.5 °C for 40 s, and at 72 °C for 40 s, with annealing temperature decreasing 0.5 °C per cycle; the programme was completed by 23 additional cycles with annealing temperature 61 °C and a final extension step at 72 °C for 10 min. The amplified DNA fragments were digested by Restriction Fragment Length Polymorphism(RFLP) by the use of specific Restriction endonuclease “NalII” (NEBiolabs) at 37 °C for 4 h [27]. Agarose gel (Metaphor ™, Rockland, ME USA) of 3% was prepared for detection of G6PD variants, where 10 µl of DNA was loaded in the gel and allowed to run at 100 V for 1.30 h. Individuals were characterized as GG (normal), AG (heterozygous) and A−/AA (hemi/homozygous).

The genotype frequency and allele frequency in the population was calculated based on the following formula:

$${Genotype}\; {frequency}\;(\%) = {heterozygous}\; {individuals} + {homozygous}\; {individuals}/{total}\; {no.}\; {of}\; {individuals}\; {genotyped}.$$

$${Allele}\; {frequency}\; (\%) = (2{\text{X}}\; {\text{homozygous}}\; {\text{female}} + {\text{hemizygous}}\; {\text{male}} + {\text{heterozygous}}\; {\text{female}})/(2{\text{X}} \; {\text{female}}\; {\text{tested}} + {\text{male}}\; {\text{tested}}).$$

### Assigning G6PD genotype to phenotype

The enzymatic activity to assign the phenotypes according to severity and clinical manifestation as per WHO recommendations [45] was not assessed. The phenotype in this study was deduced based on the genotype of a molecular detection of the G6PDA− G202A allele. The phenotype assigned were G6PD B (wild type), G6PD A (non-deficient type) and G6PD A− (deficient type).

### PCR for molecular detection of Plasmodium species

Nested PCR was used for molecular detection of *Plasmodium* species as described [46, 47].

Briefly, a nested PCR was used to amplify species-specific sequences of the small sub-unit ribosomal ribonucleic acid (18S SSU rRNA) genes of *P. falciparum*. DNA from the culture of *P. falciparum* (3D7strain), DNA from blood samples of an individual never exposed to malaria, and PCR water were used as positive and negative controls and were included in each set of PCR for quality control. The PCR was carried out in a thermo-cycler (PTC-0240, The DNA engine® Thermal Cycler, Bio-Rad, Hercules, USA), followed by gel electrophoresis to determine whether blood sample was individuals as either parasite positive or negative.

### Statistical analysis

Data were analysed using STATA (StataCorp, Texas, USA) software version 15 to generate the frequency and association tables. Frequency of parasite carriage was calculated as the sum of the number of individuals that were observed to be parasite positive over the total number of individuals. For categorical variables, Chi-squared or Fisher’s exact tests were used to assess significant differences in proportions when appropriate. All reported *p*-values are two-sided and were considered statistically significant if < 0.05. Logistic regression was used to fit analyses association of parasite carriage estimated by PCR (dependent variables) and G6PD genotype (independent variable). The relationship between G6PD genotype and phenotype was obtained using crosstabs descriptive statistics. Statistical significance was set at *p* value ≤ 0.05.

## Results

### Study participants

A total of 263 participants were recruited in the study, and 145 (55.1%) were female. The overall median age was 14 [interquartile range (IQR): 7–31] years (Table 1). Unfortunately, further demographic data was not collected such as data on weight and haemoglobin level.

**Table 1:** Distribution of study participants based on gender and age

Village	Male		Female		Age (years)	Total	
	n	%	n	%	Median (IQR)	n	%
Budutu	32	54.2	27	45.8	17 (10–36)	59	22.4
Gambajiga	32	43.8	41	56.2	10 (6–28)	73	27.8
Kanyelele	21	37.5	35	62.5	14.5 (6–29)	56	21.3
Mwaholo	33	44.0	42	56.0	15 (6–35)	75	28.5
Total	118	44.9	145	55.1	14 (7–31)	263	100

### G6PD genotype distribution in the population

In this study, the enzymatic activity (phenotype) was not independently assessed to assign individuals either deficient or normal. However, based on genotype, individuals were assigned as normal, heterozygous, homozygous and hemizygous. Hence, with the exception of individuals who were assigned as normal, the rest of the individuals were termed as deficient although not all individuals termed as heterozygous will be deficient.

The overall G6PD deficiency in the study population was 16.4%. The study found that 8.0% (21/263) of those tested were G6PD deficiency heterozygous and 8.4% (22/263) were hemi**/**homozygous G6PD deficiency (Table 2). Among the hemi/homozygous G6PD deficiency, 54.5% (12/22) and 45.5% (10/22) were female and male, respectively.

**Table 2:** Genotype, allele frequency by village

	Tested, n (%)	Normal (B/AA), n (%)	Hetero (A–A/A–B) n (%)	Homo (A–A−)/ hemi (A−), n (%)	G6PD genotype frequency (%)	Allele frequency (%)
Budutu	59	53 (89.8)	1 (1.7)	5 (8.5)	10.2	8.1
Gambajiga	73	59 (80.8)	7 (9.6)	7 (9.6)	19.2	16.1
Kanyelele	56	48 (85.7)	5 (8.9)	3 (5.4)	14.3	9.8
Mwaholo	75	60 (80.0)	8 (10.7)	7 (9.3)	20.0	16.8
Total	263	220 (83.7)	21 (8.0)	22 (8.4)	16.4	13.2

### G6PD deficiency distribution by age group, sex and village

Among heterozygous individuals observed were found at the highest frequency (15.5%) among 5–14 years age and the highest carriage of heterozygous (18.2%) was at Mwaholo village and the lowest (3.6%) at Budutu village. However, the result reveals that there was no statistically significant difference in heterozygous G6PD deficiency carriage between villages and age (*p* > 0.05). For males hemizygote frequency was highest (17.7%) among those in age group 0–4 years and the highest carriage of G6PD hemizygote frequency (12.9%) was observed at Budutu village but all these were not statistically significant (*p* > 0.05) (Table 3). G6PD deficiency was higher (21.6%) among female compared to male (9.1%), observed difference was statistically significant (*p* = 0.007).

**Table 3:** G6PD genotype distribution among age group and village by sex

	Female							Male				
	AA		B/A−, A/A−		A−/A−		*P*-value	B		A−		*P*-value
	n	%	n	%	n	%		n	%	n	%	
Age group												
0–4	18	78.3	3	13.0	2	8.7	0.893	14	82.4	3	17.7	0.347
5–14	46	79.3	9	15.5	3	5.2		35	94.6	2	5.4	
15+	56	77.8	9	12.5	7	9.7		51	91.1	5	8.9	
Village												
Budutu	26	92.9	1	3.6	1	3.6	0.290	27	87.1	4	12.9	0.812
Gambajiga	33	73.3	7	15.6	5	11.1		26	92.9	2	7.1	
Kanyelele	30	83.3	5	13.9	1	2.8		18	90.0	2	10.0	
Mwaholo	31	70.5	8	18.2	5	11.4		29	93.6	2	6.5	
Total	120	78.4	21	13.7	12	7.8		100	90.9	10	9.1	

BA or AA, B/A or AA−, A−/A− and A− refer to normal, heterozygous, homozygous and hemizygous

### G6PD deficiency and risk factors

The findings of this study showed that age group, village and gender were not associated with carriage of both G6PD deficiency heterozygous and G6PD deficiency hemo/hemizygous (*p* > 0.05) (Table 4).

**Table 4:** G6PD deficiency and risk factors for G6PD deficiency assessed

	G6PD heterozygous		G6PD homo/hemizygous	
	Odds ratio (95% CI)	P value	Odds ratio (95% CI)	P value
Age group				
0–4	1		1	
5–14	1.19 (0.30–4.66)	0.808	0.39 (0.11–1.46)	0.163
15+^a^	0.90 (0.23–3.51)	0.876	0.72 (0.23–2.19)	0.560
Villages				
Budutu	1		1	
Gambajiga	6.28 (0.75–52.80)	0.090	1.26 (0.38–4.20)	0.710
Kanyelele	5.52 (0.62–48.95)	0.125	0.66 (0.15–2.92)	0.587
Mwaholo	7.07 (0.86–58.37)	0.070	1.24 (0.37–4.13)	0.730
Gender				
Male	–	–	1	
Female	–	–	0.64 (0.30–1.35)	0.24

^a^15 + refers to 15 years and above

### Carriage of submicroscopic *Plasmodium falciparum*

Out of 248 samples analysed for submicroscopic parasites carriage, a total of 144 (58.1%) individuals were *P. falciparum* positive by PCR whereas a total of 25 samples (10.1%) and 3 samples (1.2%) were positive for *P. malariae* and *P. ovale*, respectively. None of the samples were positive for *P. vivax*. Furthermore, 16 (6.0%) samples were positive for both *P. falciparum* and *P. malariae*. Three samples (1.2%) were positive for both *P. falciparum* and *P. ovale*. None of the samples were positive for all *P. falciparum*, *P. malariae* and *P. ovale*.

In this study, there was no significant differences on submicroscopic *P. falciparum* carriage on G6PD homo/hemizygous deficiency and gender (p > 0.05). However, individuals with G6PD heterozygous deficiency were associated with carriage of submicroscopic *P. falciparum* (*p* = 0.029) (Table 5).

**Table 5:** Carriage of submicroscopic *Plasmodium falciparum* on G6PD deficiency, age, village and gender

	Odds ratio (95% CI)	P value
G6PD heterozygous deficiency	3.64 (1.14–11.60)	0.029
Age	0.99 (0.98-1.00)	0.159
Villages	1.16 (0.91–1.49)	0.237
Gender	0.72 (0.41–1.26)	0.252
G6PD homo/hemizygous deficiency	1.04 (0.66–1.64)	0.875
Age	0.99 (0.98–1.01)	0.376
Villages	1.11 (0.87–1.41)	0.394
Gender	0.70 (0.41–1.20)	0.194

## Discussion

This study describes and reports results of a cross-sectional study conducted in four villages of Misungwi district, Mwanza region, Tanzania. The main objective of this study was to map the distribution of G6PD deficiency in the study area, which is an important aspect of malaria control and elimination using primaquine.

In this study, the enzymatic activity (phenotype) to assign individuals either deficient or normal was not independently assessed. However, based on genotype individuals were assigned as normal, heterozygous, homozygous and hemizygous. Therefore, with the exception of individuals who were assigned as normal, the rest of the individuals were referred to deficient although not all individuals termed as heterozygous will be deficient. The study reveals that the prevalence of the G6PD deficiency is 16.4%, in which genotype frequency was 8.0% and 8.4% for female heterozygous, and male hemizygous/female homozygous respectively. The overall prevalence is very similar to what has been found elsewhere in sub-Saharan Africa, where it ranged from 0 to 25% [1, 48, 49] confirming that G6PD deficiency is common in malaria endemic areas. However, the interpretation of results on heterozygous individuals to be classified as either normal or deficient should be carefully considered in this study since enzymatic assessment of individuals was not carried out.

Unlike to a study which was carried out in Nigeria where high prevalence of G6PD deficiency was particularly among children 2–5 years old [50], there was no significant difference in prevalence of G6PD deficiency between age groups in this study. The result was similar to a study carried out in Nepal [51]. The reason for this difference is unknown. However, a study in Nigeria has attributed the high frequency of G6PD deficiency in children due to a high incidence of malaria parasitaemia in the area. In the current study area, a good number of individuals were having submicroscopic parasites.

Study in this area has indicated that over half of individuals have malaria in form of submicroscopic [5]. The observations on sub-microscopic parasitaemia is consistency to other findings of the studies carried out in North East Tanzania [4, 40] and other endemic countries [24], confirming that a large number of individuals are carrying sub-microscopic parasites in a malaria endemic area. Sub-microscopic parasites have been reported to contribute to malaria transmission in individuals similar to those individuals having microscopic gametocytes [52].

In this study, heterozygous G6PD deficiency was associated with carriage of submicroscopic parasites. It has already been reported in other studies that female heterozygotes are protected from severe malaria [53, 54], which can be explained by the fact that *P*. *falciparum* development is hindered in G6PD deficient red cells [24], slowing the rate of parasite replication and reducing the likelihood of severe disease [55]. However, the findings of this study on the association between G6PD deficiency and submicroscopic parasite carriage differs to a study carried out in Ghana where they found that G6PD deficiency was associated with a lower carriage of asymptomatic *P. falciparum* carriage in children aged between 6 and 12 years [56]. From the findings whether females are protected by G6PD deficiency is uncertain due to variation in study design, G6PD and to genetic differences between populations.

This high prevalence of G6PD deficiency in the area raises concern when planning for MDA for clearing of the malaria parasites in endemic areas using primaquine if a higher primaquine dose is considered [8, 57–60]. In the perspective of malaria elimination, the use of primaquine which is active against all liver stages of *Plasmodium*, and also offers activity against *P. falciparum* gametocytes, thereby blocking transmission to mosquitoes should be considered [58, 61].

Although studies in different malaria endemic countries including Tanzania [32, 62] have shown that SLDPQ is well tolerated and safe in G6PD deficiency it should be noted that higher PQ dosage are more effective and needed as indicated in Brazil [42] and Uganda [63]. Nevertheless, PQ at low dosage has been proved to be safe and successful when used as a partner drug to ACT [10, 31], but also it cannot be ignored that the proposed higher PQ dosage is associated with the risk of acute haemolytic anaemia [10, 64].

Tanzania is focusing on reducing malaria transmission and exploring the possibilities towards the malaria pre-elimination and elimination [38], the use of primaquine (SLDPQ or higher primaquine) dose will be an option. Therefore, studies on mapping G6PD deficiency in malaria hotspot area for MDA, taking into consideration of the dosage of PQ, would have provided further insight of G6PD deficiency in malaria hotspot area and help implementation of MDA [61]. Identification of remaining high-risk areas may become more important to understand G6PD distribution in other parts of the country which is very important for future malaria control and elimination and allowing resources to be targeted to areas that remain at high risk of malaria transmission.

This study has some limitations. The enzymatic activity was not assessed, haemoglobin level and other polymorphisms in the study area, and this is a topic which warrants future research.

## Conclusion

G6PD deficiency is prevalent and spatially heterogeneous across in the study area. Likewise, submicroscopic infections, which are possible infectious reservoirs of parasites, are common in the area. The government of Tanzania is implementing recommended preventive and curative interventions to malaria with the possibility of moving towards the malaria elimination. One possibility is the use of primaquine, although SLDPQ is well tolerated and safe in G6PD deficiency, a higher PQ dosage may be needed. Higher dosage of PQ is associated with acute haemolytic anaemia in G6PD deficient individuals. Awareness is needed regarding the use of a higher dose of primaquine for G6PD deficiency individuals if required.

## Data Availability

The datasets in this study are available from the corresponding author on reasonable request.

## Abbreviations

DHS: Demographic and Health Survey
G6PD: Glucose-6-phosphate dehydrogenase
HRP2: histidine-rich protein 2
MDA: Mass Drug Administration
RDT: Malaria rapid diagnostic test
SNP: Single nucleotide polymorphism
PCR: Polymerase chain reaction
